# Addressing Underrepresented Factors in Cardiovascular Mortality Trends Among Chronic Kidney Disease Patients: A Call for Comprehensive Intervention Strategies

**DOI:** 10.1002/clc.70199

**Published:** 2025-08-27

**Authors:** Shaher Yar, Zahin Shahriar, Sumaiya Ahmed, Muhammad Shehzad Asif

**Affiliations:** ^1^ University College of Medicine and Dentistry Lahore Pakistan; ^2^ Dhaka Medical College and Hospital Dhaka Bangladesh; ^3^ Department of Public Health North South University Dhaka Bangladesh

To the Editor,

I read with great interest the comprehensive retrospective analysis by Ahmad et al. examining cardiovascular mortality trends in chronic kidney disease (CKD) patients from 1999 to 2020 [1]. While the authors excellently document demographic disparities and temporal patterns in cardiovascular mortality, their work highlights several critical gaps that warrant further discussion, particularly regarding the underutilization of evidence‐based interventions and the impact of emerging therapeutic paradigms on cardiovascular outcomes in CKD patients.

## 1 Underrepresentation of Novel Cardio‐Renal Protective Therapies

The study period (1999−2020) captures a transformative era in CKD management, yet the authors do not adequately address how the introduction of novel therapies may explain certain mortality trends. The 2024 KDIGO guidelines now emphasize SGLT2 inhibitors as cornerstone therapy, showing remarkable cardiovascular benefits in CKD populations [2]. Recent meta‐analyses demonstrate that SGLT2 inhibitors reduce major adverse cardiovascular events by 9%−14% in CKD patients, with particularly strong effects on heart failure hospitalization and cardiovascular death [3].

Similarly, the emergence of non‐steroidal mineralocorticoid receptor antagonists (MRAs) like finerenone has revolutionized CKD‐cardiovascular care. The pooled FIDELITY analysis demonstrated a 14% reduction in cardiovascular death, myocardial infarction, stroke, and heart failure hospitalization, with a 23% reduction in CKD progression [4]. These therapeutic advances, introduced toward the end of the study period, likely contributed to the stabilization of mortality rates observed by Ahmad et al.

## 2 Healthcare Access and Implementation Barriers

The significant racial and geographic disparities documented by the authors reflect deeper healthcare access issues that extend beyond demographic risk factors. Research demonstrates that up to 98% of people with kidney failure in low‐income regions cannot access kidney replacement therapy, compared to 30% in high‐income countries [5]. Within the United States, these disparities manifest as differential access to nephrology care, with rural and minority populations experiencing delays in specialist referral and reduced access to evidence‐based therapies [6].

The authors' observation that non‐metropolitan areas exhibited higher age‐adjusted mortality rates (8.6 vs. 8.1 per 100 000) underscores the critical role of healthcare infrastructure in cardiovascular outcomes. Studies show that less than one‐third of community healthcare settings in resource‐limited areas can access essential diagnostics for CKD monitoring, further contributing to delayed intervention and poor outcomes [5].

## 3 Missing Context: COVID‐19 and Cardiovascular Risk Amplification

The study period concludes in 2020, capturing the early COVID‐19 pandemic impact. Recent evidence demonstrates that COVID‐19 significantly amplifies cardiovascular risk in CKD patients, with a twofold increased risk of cardiovascular death within 30 days and a 64% increased risk overall compared to non‐CKD patients. This pandemic effect may have influenced the mortality trends observed in 2020 and represents an important confounding factor not addressed in the analysis.

## 4 Implications for Clinical Practice and Future Research

The stable mortality trends observed by Ahmad et al. contrast sharply with the declining cardiovascular mortality in the general population, suggesting that CKD patients are not benefiting equally from advances in cardiovascular care. This disparity emphasizes several urgent priorities:

First, implementation science research is needed to optimize the delivery of evidence‐based therapies. Studies show that combination therapy with SGLT2 inhibitors, MRAs, and RAAS inhibitors can provide additive cardiovascular protection, yet uptake remains suboptimal.

Second, targeted interventions for high‐risk populations identified by the authors are essential. The significantly higher mortality rates in Non‐Hispanic Black or African American patients (15.37 per 100 000) demand culturally adapted cardiovascular risk reduction programs and improved access to specialty care.

Third, the healthcare system must address the fundamental infrastructure gaps that perpetuate geographic and racial disparities in cardiovascular outcomes among CKD patients.

## 5 Conclusion

Ahmad et al. provide valuable epidemiological insights into cardiovascular mortality trends in CKD patients. However, the stable mortality rates they document, contrasting with improvements in the general population, underscore the urgent need for comprehensive intervention strategies that address therapeutic underutilization, healthcare access barriers, and systematic implementation of evidence‐based care. Only through such multifaceted approaches can we hope to improve cardiovascular outcomes for the millions of Americans living with CKD.

## Author Contributions

Dr. Shaher Yar conceived, wrote, and revised the manuscript. Dr. Zahin Shahriar validated, edited, & compiled the manuscript. Dr. Sumaiya Ahmed reviewed the manuscript. Dr. Muhammad Shehzad Asif supervised the research.

## Conflicts of Interest

The authors declare no conflicts of interest.

## Data Availability

Data is not applicable as no new data were generated or analyzed in this letter.

## References

[1] E. Ahmad , S. Ahmad , A. Naeem , et al., “Trends in Cardiovascular Mortality in Patients With Chronic Kidney Disease From 1999 to 2020: A Retrospective Study in the United States,” Clinical Cardiology [Internet] 48, no. 8 (August 2025): e70174, https://pmc.ncbi.nlm.nih.gov/articles/PMC12312204/. 40742154 10.1002/clc.70174 PMC12312204

[2] C. Baigent , J. Emberson , R. Haynes , et al., “Impact of Diabetes on the Effects of Sodium Glucose Co‐Transporter‐2 Inhibitors on Kidney Outcomes: Collaborative Meta‐Analysis of Large Placebo‐Controlled Trials,” Lancet 400, no. 10365 (November 2022): 1788–1801, https://pubmed.ncbi.nlm.nih.gov/36351458/. 36351458 10.1016/S0140-6736(22)02074-8 PMC7613836

[3] R. Agarwal , G. Filippatos , B. Pitt , et al., “Cardiovascular and Kidney Outcomes With Finerenone in Patients With Type 2 Diabetes and Chronic Kidney Disease: The FIDELITY Pooled Analysis,” European Heart Journal 43, no. 6 (November 2021): 474–484, https://pubmed.ncbi.nlm.nih.gov/35023547/. 10.1093/eurheartj/ehab777 PMC8830527 35023547

[4] K. J. Jager , C. Kovesdy , R. Langham , M. Rosenberg , V. Jha , and C. Zoccali , “A Single Number for Advocacy and Communication—Worldwide More Than 850 Million Individuals Have Kidney Diseases,” Kidney International 96, no. 5 (September 2019): 1048–1050, https://pubmed.ncbi.nlm.nih.gov/31582227/. 31582227 10.1016/j.kint.2019.07.012

[5] J. M. Norton , M. M. Moxey‐Mims , P. W. Eggers , et al., “Social Determinants of Racial Disparities in CKD,” Journal of the American Society of Nephrology 27, no. 9 (May 2016): 2576–2595, https://pubmed.ncbi.nlm.nih.gov/27178804/. 27178804 10.1681/ASN.2016010027 PMC5004663

[6] P. Rossing , M. L. Caramori , J. C. N. Chan , et al., “Executive Summary of the KDIGO 2022 Clinical Practice Guideline for Diabetes Management in Chronic Kidney Disease: An Update Based on Rapidly Emerging New Evidence,” Kidney International 102, no. 5 (October 2022): 990–999, https://pubmed.ncbi.nlm.nih.gov/36272755/. 36272755 10.1016/j.kint.2022.06.013

